# To Dr. Batty

**Published:** 1803-12-01

**Authors:** Robert Earnest

**Affiliations:** House Surgeon to the Sheffield Infirmary


### To Dr. BATTY.

SIR,

THE following communication is intended to shew the inefficacy of Digitalis Purpurea, of which so much has been said as to its specific effects in Phthisis.

After reading so many encomiums on the virtue of this well known plant, I was determined, the first convenient opportunity, to give it the fairest possible trial in two cases of phthisis pulmonalis; and this medicine was exhibited in the form of a tincture, made according to the formula recommended by Dr. Maclean, and which is inserted in the Medical and Physical Journal, vol. ii. p. 122.

The relation of the two following cases will point out the uncertain effects of this medicine, which is so generally well spoken of in diseases of this class. No doubt need arise as to the badness or impureness of the plant, as it was gathered in the beginning of July, when in its full vigour and blossom.

Before I draw up the cases, I think it necessary to remark upon what some authors have said, who have written in favour of the good effects of this plant in cases of phthisis pulmonalis. It is said, that in true cases of this disease, disease, where the following symptoms run very high, viz. hectic fever, pulse beating from 120 to 130 strokes in the minute, hæmoptysis now and then, great expectoration of purulent matter, nocturnal sweats, and sometimes diarrhœa: I say, it is said that in such cases the digitalis is capable of effecting the cure of such a malady; and which is performed by these means: It diminishes the frequency of the pulse, of course the fever must be abated, as also the hæmoptysis if present; it gradually checks the expectoration, consequently the bowels are less liable to be out of order, "as I am firmly of opinion, that it is in the matter swallowed from time to time that in a general way brings on this spontaneous kind of diarrhœa, as I have many times found that the stools of such patients consist of similar matter to that which has been coughed up from the lungs. All these symptoms are gradually made better, the patient begins to recover his strength, of course the night sweats disappear, and in the end he is completely cured."

I sincerely wish the two following cases would strengthen the above declaration; but am extremely sorry to say, that they prove very much to the contrary. I attended the patients myself, and made very accurate minutes, morning and evening, for three weeks successively, both of the state of the pulse, the quantity and quality of the matter expectorated, and the quantity of tincture given morning (ten o'clock), and afternoon (four o'clock).*

Case I. Thomas Wilkinson, æt. 33, by trade a grinder, of a spare habit of body, of the middle stature, a delicate and florid complexion. He was admitted into the Sheffield General Infirmary on March 6, 1801, and was then labouring under the following symptoms, viz. A hectic fever; the pulse beating 120 strokes in the minute; a troublesome cough and great expectoration, to the amount of from four to five ounces of purulent matter in the course of twenty-four hours; night or rather morning sweats, so as to wet his linen; in very hot or cold weather, hæmoptysis would come on, and continue a few days; sometimes he had a diarrhœa; debility, and loss of appetite.

This

* Dr. Maclean says, that from ten to thirty drops of his tincture, ter indies, is a sufficient dose, but I extended it a little further, and only bis indies, as I found by experience that when it was given so frequent as three times in the day, that it was the cause of keeping the stomach in a state of continual nausea. Pyrmont water was the vehicle which I gave the tincture in.

This appeared a fair case for the trial of digitalis; and after clearing the stomach and bowels, he was, on the 12th of March, put upon this indication of cure, which he continued regularly for three weeks, without the least benefit, as may be seen from the annexed table of minutes, made at the times of visiting.

| Dates of visiting. | State of the pulse morn | State of the pulse even. | Quantity of tincture given morning. | Quantity of tincture given evening. | Quantity of matter expectorated. | Quality of the matter. |
|---|---|---|---|---|---|---|
| Mar. 12 | 120 | 120 | drops 5 | drops 5 | about 5 oz. | pus |
| 13 | 120 | 120 | 6 | 6 | ditto | ditto |
| 14 | 120 | 120 | 7 | 7 | ditto | ditto |
| 15 | 120 | 120 | 10 | 10 | ditto | ditto |
| 16 | 112 | 112 | 12 | 12 | ditto | ditto |
| 17 | 120 | 120 | 15 | 15 | ditto | bloody |
| 18 | 120 | 108 | 18 | 18 | less | ditto |
| 19 | 120 | 120 | 21 | 21 | ditto | less so |
| 20 | 124 | 124 | 24 | 24 | more | pus |
| 21 | 124 | 124 | 28 | 28 | ditto | ditto |
| 22 | 124 | 124 | 30 | 30 | ditto | ditto |
| 23 | 124 | 124 | 33 | 33 | ditto | ditto |
| 24 | 124 | 124 | 36 | 36 | ditto | ditto |
| 25 | 124 | 124 | 38 | 38 | ditto | ditto |
| 26 | 124 | 124 | 40 | 40 | more | ditto |
| 27 | 124 | 124 | 42 | 42 | ditto | ditto |
| 28 | 124 | 124 | 44 | 44 | ditto | ditto |
| 29 | 124 | 124 | 46 | 46 | ditto | ditto |
| 30 | 124 | 124 | 48 | 48 | ditto | ditto |
| 31 | 124 | 124 | 50 | 50 | ditto | ditto |
| *April 2 | 124 | 124 | 50 | 50 | ditto | ditto |
| 3 | 124 | 124 | 50 | 50 | ditto | ditto |
| 4 | 124 | 124 | 50 | 50 | ditto | ditto |

CASE II. William Stokes, æt. 16, rather tall of his age, was of a plethoric habit, and had been ailing some time with pulmonic affections, attended with a cough and glandular obstructions about the neck. He was born of healthy parents

* On account of some aperient medicine that was given on the 1st of April, he omitted taking the tincture, and after the 4th he left it off altogether, not being at all relieved by it, as may be seen above; he however has been relieved by various other remedies; and continued lingering in this way, better and worse, until the beginning of April last, when his symptoms returned with more violence, and in the course of that month he died.

parents on the mother's side, but his father died some years ago of phthisis; his mother still lives, and is a strong, healthy woman.

He was admitted into the Sheffield General Infirmary, July, 1801, on account of the disease in his neck; at the same time he had the symptoms of phthisis, such as hectic fever, a small, quick pulse, beating from 125 to 130 strokes in the minute; coughed and spit up daily a great quantity of pus, which was sometimes streaked with blood; had night sweats, wandering pains in the thorax and about the sides, great debility, and loss of appetite. With these symptoms he was presently reduced from a plethoric state to that of an emaciated one. Various remedies were employed for some time, in order to lessen or subdue the symptoms, but all to no lasting relief; in the end, he was put upon the digitalis.

He began and continued the use of it exactly in the same manner for three weeks, as Wilkinson did, without the least variation, either in the pulse or the other symptoms; † at the expiration of the time above mentioned, he left off the digitalis, and was put upon palliatives, &c. for the relief of the cough and other symptoms. He continued gradually declining, until the July following, 1802, when he died.

In many other cases of phthisis, I have frequently remarked the disuse of digitalis.

I am, &c.

*November* 12, 1803. ROBERT EARNEST,

House Surgeon to the Sheffield Infirmary.

---

### *Observations on the Utility of amputating the Extremities of the Bones in Cases of Caries of the Joints; by* M. MOREAU, M. D. *Communicated by our Correspondent at Paris.*

THE Author details a number of Cases where this mode of treatment superseded the necessity of amputating the entire extremity, and even without any considerable defect

---

† Although the symptoms were not relieved, in either case, both the patients most assuredly were under the influence of the medicine, as they many times complained of nausea, vertigo, languor, and a loathing of their diets.

